# Ulcerative Necrobiosis Lipoidica in the Setting of Epstein-Barr Virus-Positive Diffuse Large B-Cell Lymphoma and Glucose-6-Phosphate Dehydrogenase Deficiency

**DOI:** 10.7759/cureus.98791

**Published:** 2025-12-09

**Authors:** Mary J Casey, Francesca L Veon, Anisha Bhanot, Brad Gehrs, Jeffrey D McBride

**Affiliations:** 1 College of Medicine, University of Oklahoma Health Sciences Center, Oklahoma City, USA; 2 Mark Allen Everett Department of Dermatology, University of Oklahoma Health Campus, Oklahoma City, USA; 3 Department of Pathology, University of Oklahoma Health Campus, Oklahoma City, USA; 4 Department of Pathology and Laboratory Medicine, Oklahoma City VA Medical Center, Oklahoma City, USA

**Keywords:** diffuse large b-cell lymphoma, epstein barr virus positive, glucose-6-phosphate dehydrogenase deficiency, granulomatous reaction, lymphoma, necrobiosis lipoidica

## Abstract

Necrobiosis lipoidica (NL) is a chronic granulomatous disease most commonly manifesting as yellow, atrophic plaques and ulcerations on the lower extremities. We present an exacerbation of NL in a patient with a known history of glucose-6-phosphate dehydrogenase deficiency, who, following a clinical and histopathologic diagnosis of NL, was found to have oropharyngeal Epstein-Barr virus-positive diffuse large B-cell lymphoma. After implementation of therapies for lymphoma, chemotherapeutic complications, and NL, the patient reported improvement in both pain and ulcerations. Though noninfectious granulomatous reactions are common in lymphoproliferative disorders, the skin is rarely involved. This case presentation provides evidence for an association between NL and underlying lymphoma development and encourages clinicians to rule out lymphomatous processes in the setting of reoccurring or recalcitrant noninfectious cutaneous granulomas.

## Introduction

Necrobiosis lipoidica (NL) is a rare, chronic granulomatous disease commonly presenting as erythematous papules and nodules that coalesce into telangiectatic, atrophic plaques and ulcerations on the anterior aspect of the lower extremities [1]. Other areas of the body such as the upper extremities, groin, torso, and face can be involved, but are less common [1,2]. The majority of presentations are painless, with pain occurring primarily when ulcerations are present. NL predominantly occurs in women with a reported range of 3:1-5:1 female-to-male ratio and typically arises in the third to fourth decades of life [1,3]. Pathological features include a swelling of blood vessel endothelium and granulomatous inflammation that spans the entire depth of the dermis, presenting in a layered, horizontal distribution, which is separated by necrobiotic collagen [4]. This presentation is commonly described as a “cake layer” pattern.

The etiology of NL is unclear, with leading theories for pathogenesis including microangiopathy, deposition of immunoglobulins, metabolic influences, and abnormal collagen production as potential causative agents [1,2]. NL has been most notably associated with diabetes mellitus; other proposed associations include autoimmune thyroiditis, rheumatoid arthritis, obesity, hypertension, and hyperlipidemia [3,5]. First-line treatment includes intralesional, topical, and occasionally systemic corticosteroids [5,6]. Recently, phototherapy, biologics, immunomodulators, and JAK inhibitors have been shown to further improve outcomes for patients [5,6].

This case report presents an exacerbation of NL in the rare setting of a history of glucose-6-phosphate dehydrogenase (G6PD) deficiency and a concurrent diagnosis of Epstein-Barr virus (EBV)-positive diffuse large B-cell lymphoma (DLBCL). This report supports a possible association with recalcitrant NL and underlying lymphoma, describes treatment in adjunct with co-occurring oncology interventions and G6PD deficiency considerations, and describes NL outcomes under this patient’s unique treatment course.

This work was previously presented as a poster at the 2025 University of Oklahoma College of Medicine Research Symposium on October 17, 2025.

## Case presentation

A 60-year-old Hispanic male with G6PD deficiency presented to the dermatology clinic in August of 2024 with a primary concern of worsening, painful ulcerations of the lower extremities. Previous failed treatments included pentoxifylline, etanercept, apremilast, infliximab, and unsuccessful surgical excision several years prior. A course of prednisone initiated in February 2022 was the only treatment reported to improve the patient’s pain and presentation.

**Figure 1:**
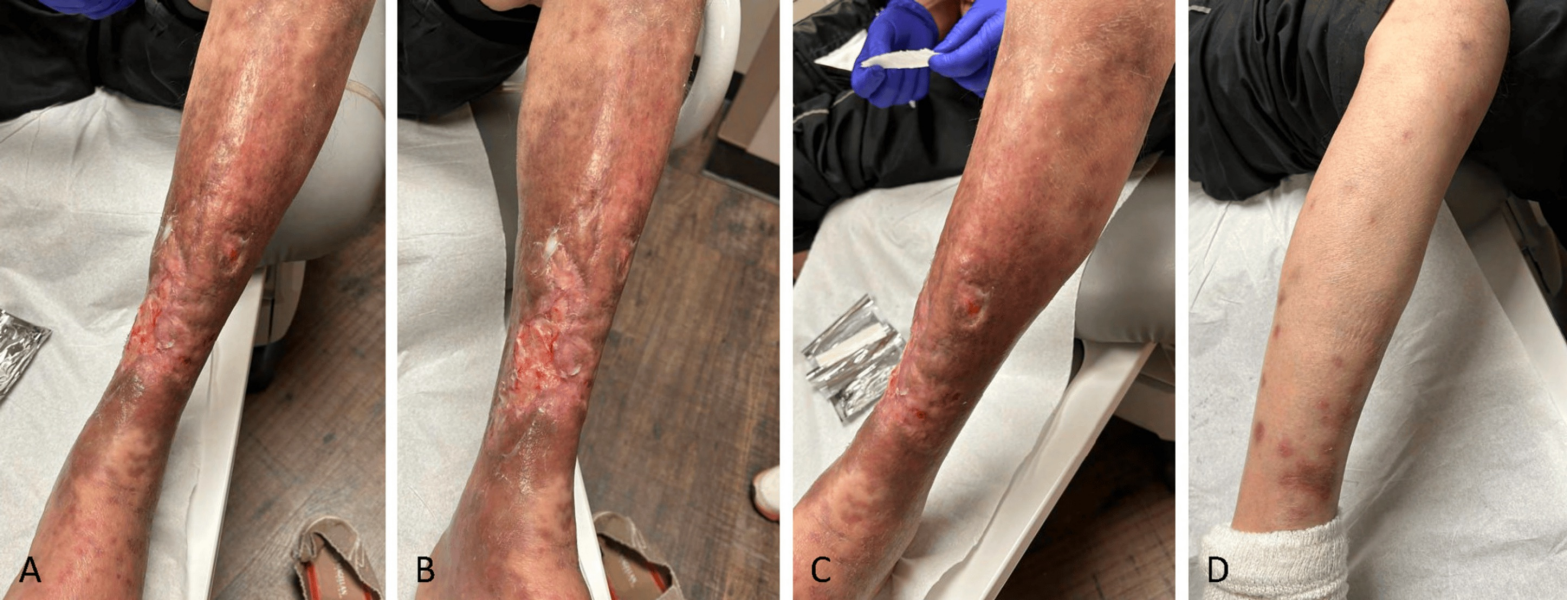
Ulcerated plaques and hyperpigmented macules showing necrobiosis lipoidica characteristics. A-C. Left shin coalescing ulcerations with granulation tissue. There were surrounding skin atrophy and hyperpigmentation (A: medial, B: anterior, C: lateral). D. Right leg hyperpigmented macules and papules with slight scale, atrophy, overlying erythema, and regions of coalescence.

**Figure 2:**
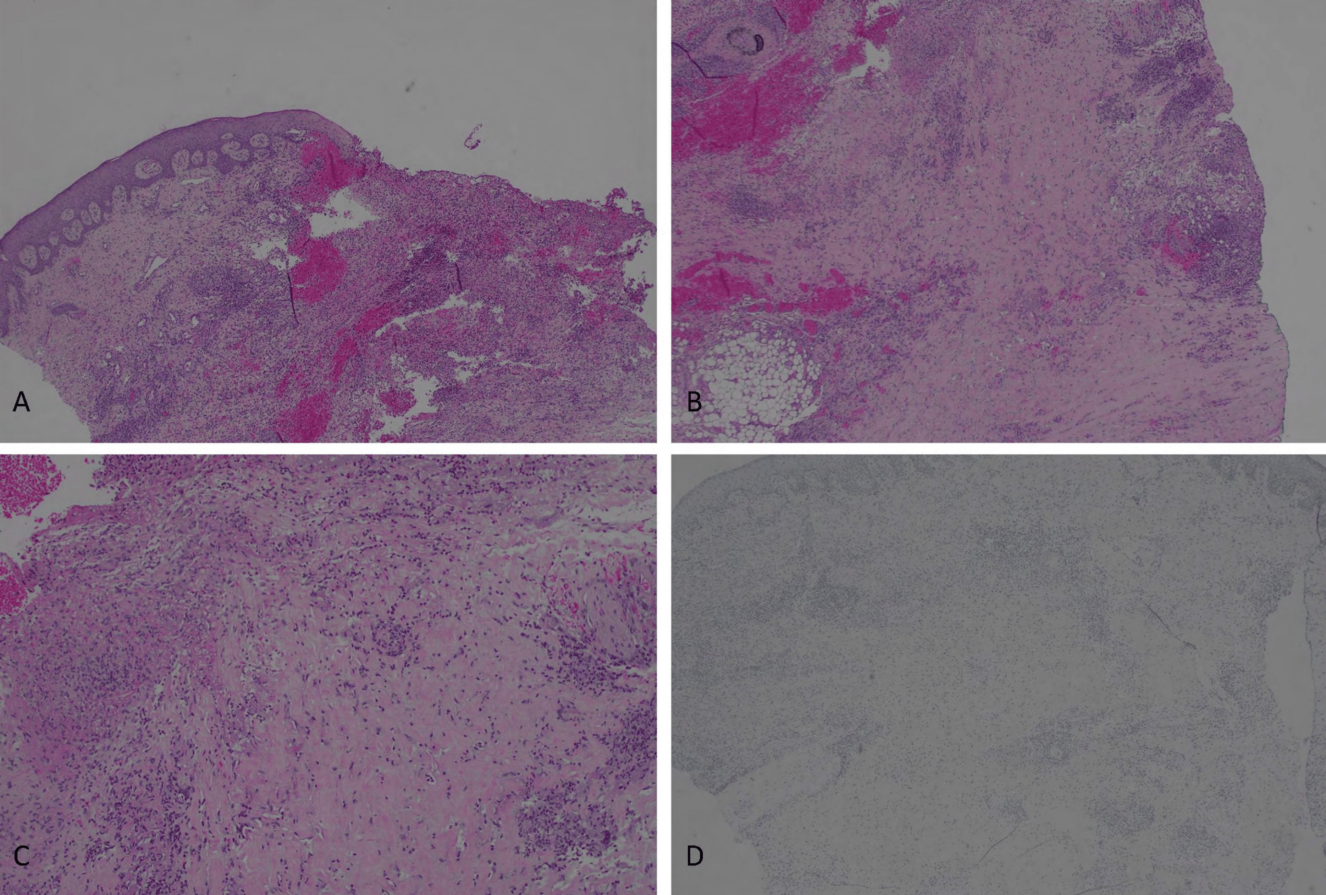
Cutaneous ulcer with features of necrobiosis lipoidica. A. Ulceration with layered histiocytes and lymphocytes (40X). B-C. Histiocytes and lymphocytes with areas of necrobiotic collagen (B=40X, C=100X). D. CD20 is negative within the vast majority of lymphocytes (indicating negative for B-cell lymphoma).​ Note for the methods: magnification is objective magnification x lens magnificent (10X).​

**Figure 3:**
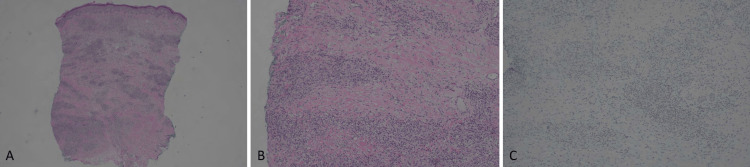
Right upper arm nodules with necrobiosis-like histology. A. Sections reveal layered histiocytes and lymphocytes with bands of necrobiotic collagen (20X). B. Higher power revealing the layered appearance of histiocytes and lymphocytes (100X). C. CD20 was negative in the nodules, arguing against a B-cell lymphoma (100X).

The physical exam demonstrated small, ulcerated plaques with coalescing larger, elliptical-shaped ulcerations. Visible granulation tissue on the left shin was present, as well as numerous hyperpigmented macules and papules scattered on the right lower extremity. The patient also began to develop nodules with overlying erythema (Figure 1). A punch biopsy of multiple lesions (including periwound skin and non-ulcerated nodules) showed dense histiocytic/granulomatous dermal infiltrates with some CD3+ T cell lymphocytic infiltrates and some lipomembranous changes within adipocytes. There were very sparse CD20-positive B cells (Figure 2, Figure 3). These findings resembled an NL-like process, and with clinical correlation, NL was diagnosed. 

**Figure 4:**
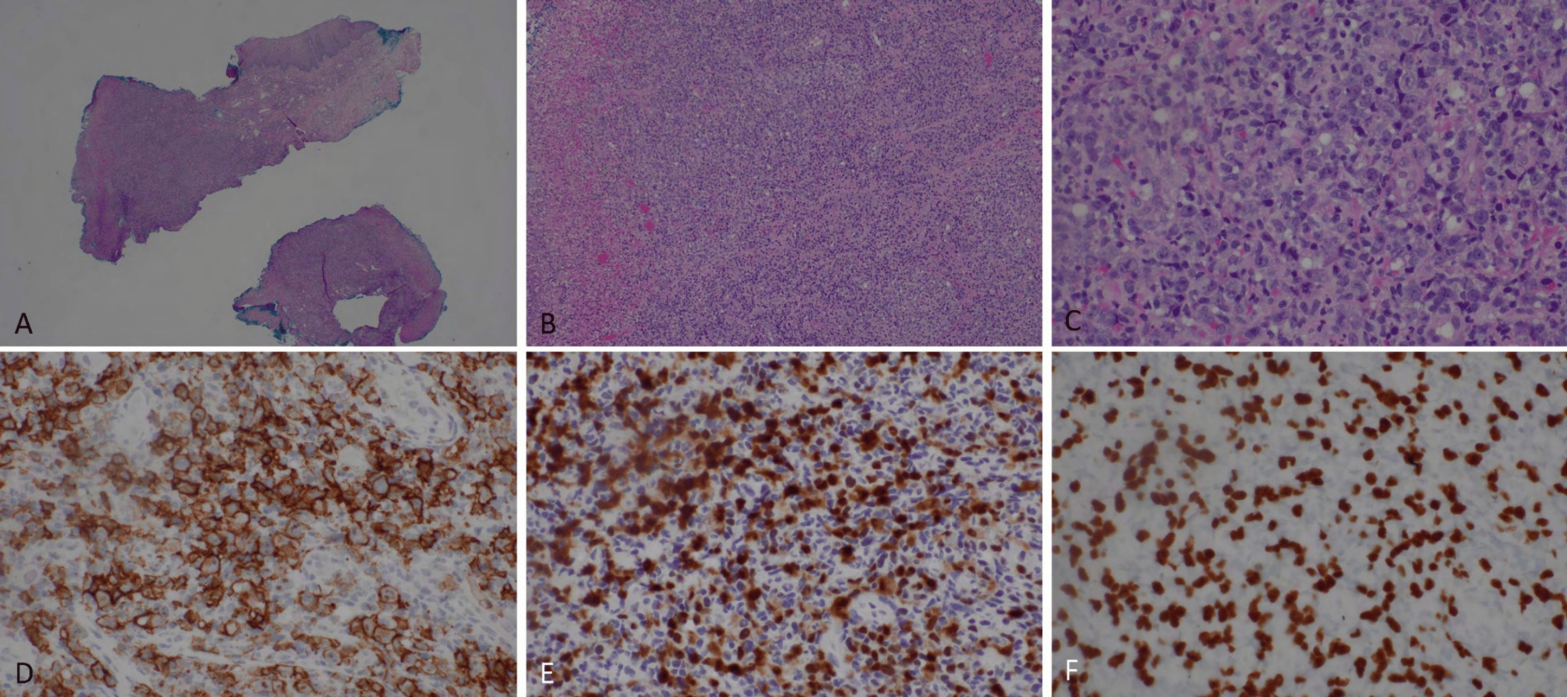
Oropharyngeal EBV+ large B-cell lymphoma. (A: 40X, B: 100X, C: 400X). D. Mass contained diffuse CD20+ B-cells (400X). The B-cells were also positive for MUM1 (E: 400X) and EBV (F: 400X).

At the follow-up in October 2024, three lesions on the left anterior shin were injected with 0.7cc of intralesional triamcinolone 10mg/ml. Additionally, the patient was given clobetasol 0.05% ointment and a hypochlorous acid solution for wound care of NL ulcerations. Shortly thereafter, in the same month, the patient was admitted to the hospital for treatment of a right middle meatus infection associated with odynophagia, poor oral intake, and acute unintentional weight loss. During this inpatient stay, a biopsy of the soft palate revealed EBV-positive DLBCL (Figure 4). Following his first two cycles of a chemotherapy regimen including rituximab, cyclophosphamide, doxorubicin, vincristine, and prednisone (R-CHOP), he developed methicillin-resistant Staphylococcus aureus and Pseudomonas bacteremia which required inpatient IV antibiotics. At this time, dermatology also modified the patient’s topical regimen to include gentamicin 0.1% ointment and mupirocin 2% ointment due to secondary infection. Additionally, the initiation of R-CHOP treatments contributed to significant neutropenia and thrombocytopenia which caused a temporary halt of R-CHOP cycles. The patient was given pegfilgrastim and platelet infusions to address these manifestations. 

At a dermatology follow-up appointment in February 2025, the patient reported improvement in both pain and ulceration after the implementation of not only dermatologic and antimicrobial treatments, but also R-CHOP chemotherapy and interventions for cytopenia. Therefore, the improvement could be a result of dermatologic treatment, DLBCL systemic therapy, or multifactorial. His dermatologic treatment plan of gentamicin 0.1% ointment, mupirocin 2% ointment, clobetasol 0.05% ointment, and a hypochlorous acid solution for wound care was continued. Notably, dapsone was avoided due to known G6PD deficiency as well as other systemic immunosuppressants due to current DLBCL and planned continuation of R-CHOP chemotherapy. 

## Discussion

Current proposed associations with NL include metabolic and autoimmune disorders such as diabetes, autoimmune thyroiditis, rheumatoid arthritis, obesity, and hyperlipidemia [3,5]. We present a case of a 60-year-old man diagnosed with EBV-positive DLBCL of the oropharynx following presentation to dermatology for exacerbation of NL skin lesions. Though concomitant NL with DLBCL is rare, noninfectious granulomatous reactions are known amongst various lymphoproliferative disorders. Therefore, we propose that our patient’s cutaneous exacerbation of NL was associated with the underlying oropharyngeal DLBCL development and describe the possibility of NL arising in the setting of lymphoma.

The evolution of noninfectious granulomas is common in lymphoma; however, the skin is rarely involved. There are two types of cutaneous granulomas identified in the setting of lymphoma. The first type is granulomatous infiltrates with the presence of neoplastic cells (which our patient did not have, as cutaneous biopsies lacked malignant B-cells), indicating involvement of malignant lymphoma. The second type describes granulomatous skin manifestations that have no neoplastic cells (consistent with our patient) [7]. Due to the absence of lymphoma-derived cells, diagnosing underlying lymphomas is more challenging, especially from a histopathologic perspective. Typically, granulomatous processes in organs without lymphoma involvement occur more frequently in lymph nodes, spleen, and liver [7]. Our case of oropharyngeal DLBCL diagnosis following a cutaneous NL aligns with the second type of manifestation, which occurs in the skin less frequently.

Only one previous case report from 2018 has reported NL presentation before the diagnosis of an underlying lymphoma. Though this report does not discuss NL presentation after interventions or therapeutic decisions beyond the initiation of chemotherapy, the authors propose that transformation of an indolent lymphoproliferative disorder into a more aggressive lymphoma could be associated with their patient’s NL recurrence, possibly via collateral activation of histiocytes as a byproduct of reaction to underlying malignancy [7].

## Conclusions

This case of a 60-year-old male patient diagnosed with EBV-positive oropharyngeal DLBCL following an NL exacerbation describes the improvement of NL presentation after chemotherapeutic, dermatologic, and antimicrobial treatments. Diagnosis of an underlying lymphoma dramatically shifted the therapeutic approach away from immune or histiocytic suppression towards chemotherapy targeting the underlying malignancy. Medications contraindicated in G6PD deficiency, most notably dapsone, were also avoided. Clinicians should be encouraged to garner a high degree of suspicion for the association of lymphomatous processes underlying reoccurring or recalcitrant noninfectious cutaneous granulomas, even despite lacking overt neoplastic cell involvement in the skin. Furthermore, dermatologists should consider ruling out underlying malignancy with any new onset of seemingly spontaneous NL-like lesions or ulcerations.
